# Chromosome-level genome assembly of *Ajuga decumbens*

**DOI:** 10.3389/fpls.2024.1413468

**Published:** 2024-06-19

**Authors:** Yubang Gao, Jingzhao Li, Yuli Xie, Teng Zhang, Kai Tian, Xiaotang Li, Lunguang Yao

**Affiliations:** ^1^ School of Life Sciences and Agricultural Engineering, Nanyang Normal University, Nanyang, Henan, China; ^2^ Henan Province Artemisia Argyi Development and Utilization Engineering Technology Research Center, Nanyang Normal University, Nanyang, Henan, China; ^3^ Henan Field Observation and Research Station of Headwork Wetland Ecosystem of the Central Route of South-to-North Water Diversion Project, School of Life Sciences and Agricultural Engineering, Nanyang Normal University, Nanyang, Henan, China

**Keywords:** *Ajuga decumbens*, Chinese herbal medicine, genome assembly, terpene synthase, HiFi sequencing

## Introduction

**Figure 1:**
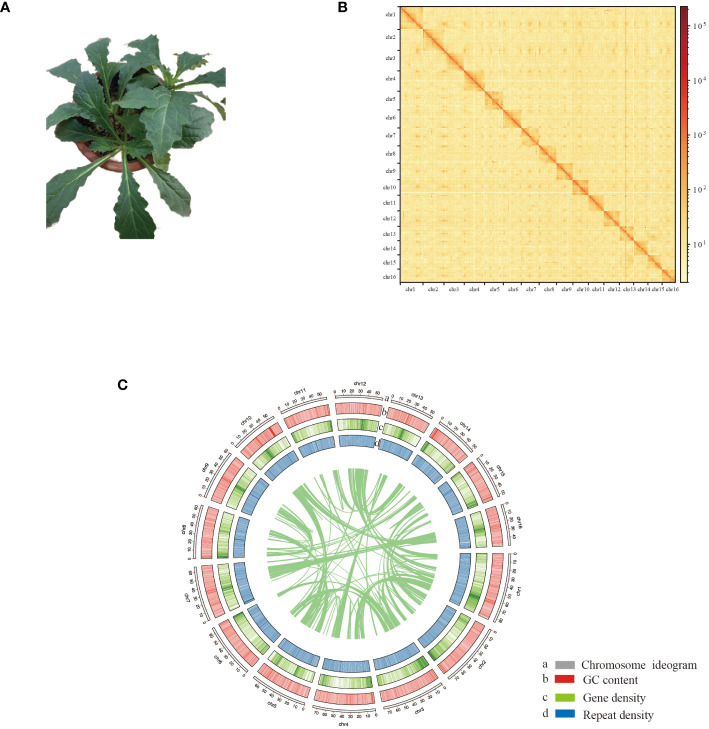
Chromosome-scale assembly of the *A. decumbens* genome. **(A)** The phenotype of the sequenced plant. **(B)** The Hi-C interaction heatmap shows 100-kb resolution super scaffolds. **(C)** Features of *A. decumbens* genome. a: Length of each pseudochromosome (Mb). b: Distribution of the GC content. c: Distribution of gene density. d: Distribution of repetitive sequence.

*Ajuga decumbens*, a critical species in the Ajuga genus, is a flowering plant widely used in traditional Chinese medicine (**Figure 1A**). The Ajuga genus encompasses more than 300 plant species. It is chiefly found in temperate regions across Europe, Asia, Australia, North America, and Africa (Atay et al., 2016; Park et al., 2017). Prominent species in this genus are recognized for their medicinal properties (Qing et al., 2017). Phytochemical studies have identified a range of bioactive compounds in Ajuga, such as phytosterols, diterpenoids, triterpenoids, sesquiterpenoids, and iridoids (Qing et al., 2017; Dong et al., 2020). Pharmacological studies have shown that Ajuga has anticancer (Graham et al., 2000), antipyretic (Shafi et al., 2004; Debell et al., 2005), anti-inflammatory (Korkina et al., 2007; Dong et al., 2020; Liu et al., 2020), antioxidant (Chenni et al., 2007; Bouderbala et al., 2008), anti-malarial (Kuria et al., 2002), antimicrobial (Chen et al., 1999; Kariba, 2001), anti-arthritic (Ono et al., 2008), antitumor (Wessner et al., 1992; Cárdenas et al., 1994), anti-tussive (College, 1977), hypoglycemic (El Hilaly and Lyoussi, 2002; Chenni et al., 2007), and insecticidal properties (Jbilou et al., 2006, 2008).

Genomics plays a significant role in uncovering medicinal plants’ biological traits, chemical synthesis mechanisms, gene-assisted breeding, and synthetic biology. The development of a high-quality genome for the medicinal plant *Artemisia annua* aids in elucidating the biosynthesis of artemisinin and in studying genes highly related to its content, thereby facilitating the breeding of *Artemisia annua* and the development of drugs to combat antimalarial resistance (Shen et al., 2018; Liao et al., 2022). By analyzing the *Panax schinseng* genome, further insights into the diversity of ginsenosides have been elucidated (Kim et al., 2018). The assembly of the *Salvia miltiorrhiz*a genome revealed that tanshinones, which accumulate primarily in the roots and most abundantly in the hairy roots. Studies on the biosynthesis of phenolic acids have advanced the breeding of new varieties and biochemical research (Xu et al., 2016; Song et al., 2020; Yang et al., 2022). Therefore, obtaining high-quality genomes of medicinal plants and conducting gene mining and synthetic pathway analysis for active ingredients, coupled with strategies such as functional genomics, are crucial for the rapid advancement of medicinal plant biology.

Only the chromosome-level genome data of *Ajuga chamaepitys* is available from NCBI (GCA_958295605.1), but it lacks corresponding gene information. The widely used *A. decumbens* still lacks equivalent genome data. The high-quality genome of *A. decumbens* plays a crucial role in unraveling the genetic underpinnings of significant traits, driving genetic advancements, and aiding in synthesizing pharmacological compounds. PacBio HiFi sequencing can produce high-quality contigs, while Hi-C sequencing is instrumental in sorting and orienting these contigs (Dudchenko et al., 2017; Cheng et al., 2021). Hi-C sequencing has been successfully applied to generate high-quality genome sequences in complex organisms (Chang et al., 2023; Nakandala et al., 2023). In this study, we performed PacBio HiFi sequencing and Hi-C sequencing of *A. decumbens*, examining its high-quality genomic information at the chromosome level. This genome resource will significantly advance the exploration of medicinal resources and the cultivation of new varieties in the Ajuga genus.

## Results

### Genome sequencing and genome assembly

Previous studies estimated a genome size of 1.1 Gb (Choi et al., 2019). PacBio HiFi long reads and Hi-C sequencing data were generated (**Supplementary Table S1**). Our sequencing produced approximately 17.8 Gb of HiFi reads, resulting in a 1.1 Gb genome assembly. The assembly contains 1666 contigs with an N50 contig size of 2.09 Mb. Using the Hi-C data, we effectively organized the contigs into 16 clusters, corresponding to the 16 chromosomes of *A. decumbens*. Pseudochromosome lengths varied from 49.8 to 84.6 Mb. The Hi-C data contact matrix heatmap (**Figure 1B**) effectively demonstrated the contigs’ clustering, arrangement, and orientation. The final chromosome-level assembly of the *A. decumbens* genome was 970 Mb (**Supplementary Table S2**). 99.89% of HiFi reads were aligned to the genome. The completeness of the genome assembly was further validated by BUSCO (Benchmarking Universal Single-Copy Orthologs), indicating 99.44% coverage of essential conserved plant proteins. The k-mer QV reached 56.02%. The k-mer error rate was 2.55e-06. These results indicated that the genome was highly accurate. The genome’s raw LAI was 18.19, and the adjusted LAI was 12.56, meeting the reference genome standards. These results suggest that the genome assembly is of good quality.

### Genome annotation

Further annotation of the assembled *A. decumbens* genome revealed a high repeat content of about 76.1%, with retrotransposons (Class I elements) making up 56.92% (**Supplementary Table S3**). Long terminal repeat (LTR) sequences, including 31.54% Gypsy-type and 12.19% Copia-type, comprised 46.73% of the genome. It is similar to many other plant genomes where LTR-retrotransposons are predominant.

Additionally, we annotated protein-coding genes by integrating homology-based searches and RNA-Seq data. This hybrid approach identified 32,452 gene models in the *A. decumbens* genome. These models have an average coding sequence (CDS) length of 1,184 bp and an average of 4.59 exons per gene (**Supplementary Table S4**). Among these genes, 25,347 (78.11%) showed homology with Arabidopsis proteins, and 31,927 (98.38%) were functionally annotated using multiple public databases. **Figure 1C** demonstrates the typical complementary relationship between the density of genes and the repetitive elements in the genome. The analysis subsequently predicted non-coding RNAs in the genome, including 20,493 rRNAs, 5,029 tRNAs, 106 miRNAs, and 9,568 snRNAs.

### Phylogenetic analysis

**Figure 2:**
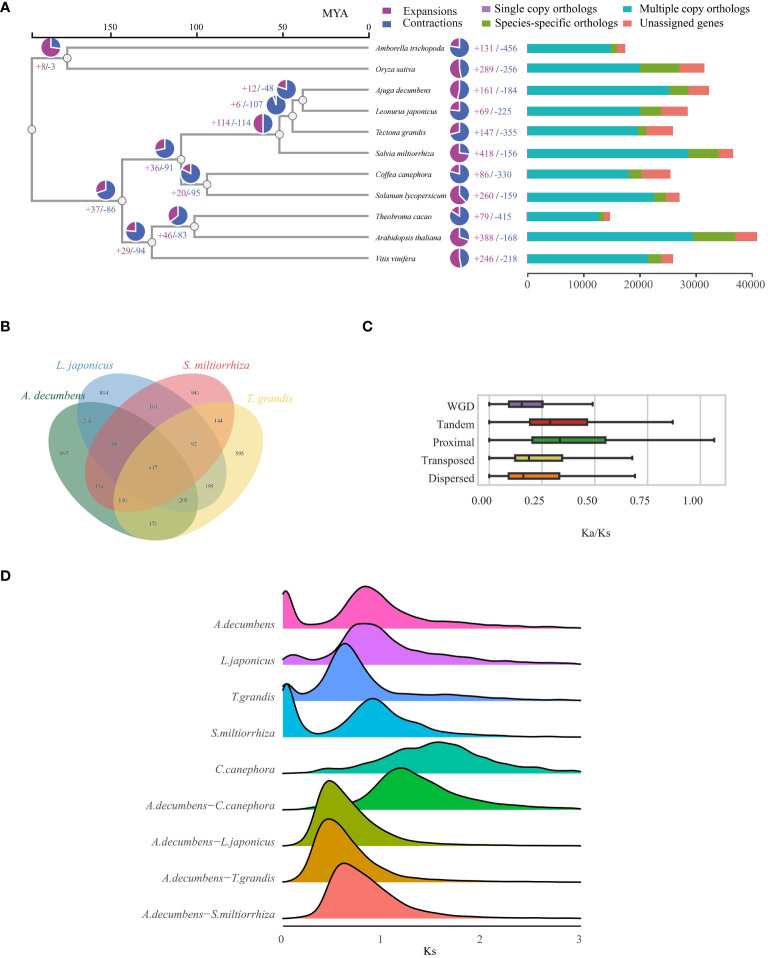
Evolutionary Analysis of the *A. decumbens* genome. **(A)** A phylogenetic tree based on shared single-copy gene families and proportion of expanded/contracted/remained gene families in each plant species (pie charts). **(B)** Venn Diagram Representation of Gene Family Overlaps and Specificities Among *A. decumbens*, *L. japonicus*, *T. grandis*, and *S. miltiorrhiza* in Labiatae. **(C)** The distribution of Ka/Ks ratios in different duplicated genes. **(D)** Distribution of Ks for gene pairs in syntenic blocks from intraspecies or interspecies genome comparison involving *A. decumbens* and different angiosperm species.

To examine the evolutionary history of *A. decumbens*, we assessed 11 angiosperm species, including a basal angiosperm (*A. trichopoda*), a monocot (*O. sativa*), and nine dicots (**Supplementary Table S5**). Analysis of these species yielded 307,695 proteins, from which 28,699 gene families were identified. Of these, 3,039 families were common across the species, indicative of a shared angiosperm ancestor. Among these gene families, 182 single-copy orthologous genes were found (**Supplementary Table S6**), aiding in constructing a phylogenetic tree. This tree showed *A. decumbens* and *L. japonicus* as closely related, having diverged 38.26 million years ago (**Figure 2A**). We pinpointed 897 gene families unique to *A. decumbens*, comprising 3,375 genes (**Figure 2B**). Of these, 2,980 genes (88.30%) were annotated. Notably, *A. decumbens* showed gene family expansion (161 families, 1,299 genes) and contraction (184 families, 232 genes), with the expanded genes being predominantly associated with salt stress resistance (31 genes).

Our comparative study of *A. decumbens*, *L. japonicus*, *T. grandis*, and *S. miltiorrhiza* identified 417 common gene families among these species. 897 gene families (3,375 genes) were unique to *A. decumbens*. We discovered 15,017 LTR retrotransposons in *A. decumbens*, with a notable burst of retrotransposition around 100,000 years ago (**Supplementary Figure S1**). The genome showed predominant Dispersed Duplication (DSD) genes, with Tandem Duplication (TD) genes also being significant (**Supplementary Figure S2**). We calculated non-synonymous (Ka) and synonymous (Ks) substitution rates and their ratios (Ka/Ks) for gene pairs from various duplication types. This revealed distinct trends in Ka/Ks ratios across duplication modes (**Figure 2C**), where TD and Proximal Duplication (PD) genes had higher ratios compared to others, especially Whole Genome Duplication (WGD) genes with the lowest ratios. The Ks distribution indicates a significant whole genome duplication (WGD) in the *A. decumbens* genome, with a prominent Ks peak at 0.8 (**Figure 2D**). Compared to *A. decumbens*, *C. canephora* lacks the γ event of whole genome duplication. Dot plots, collinearity maps, and depth charts (**Supplementary Figures S3**-**S5**) demonstrate a 2:1 collinearity relationship between *A. decumbens* and *C. canephora*. This event is widespread in the Lamiaceae family (**Figure 2D**). Additionally, PD and TD genes were significantly enriched in diterpenoid and terpenoid metabolic processes (**Supplementary Figures S6**, **S7**), underscoring their importance in *A. decumbens*’ terpene metabolism.

## Discussion

The genus Ajuga holds significant medicinal and economic value, yielding a diverse array of compounds, including phytosteroids, new iridoid and diterpene compounds, triterpenes, sterols, anthocyanin-glucosides, iridoid glycosides, solanesol, flavonoids, triglycerides, and essential oils. In recent decades, most research has centered on the pharmacology of *A. decumbens*, with genomics of the species receiving minimal attention.

The genomic research on *A. decumbens* has laid the foundation for future comparative genomics studies. It has opened new avenues for enhancing our understanding of the genetic diversity, evolutionary relationships, and functional genomics of *A. decumbens*. *A. decumbens*’ genome data assists researchers in understanding the genetic diversity within the genus, uncovering unique genetic traits that enable adaptation to diverse environments, and further exploring how these genetic variations promote phenotypic diversity and ecological adaptability. According to the CCDB database (Rice et al., 2015), the chromosome numbers in the genus Ajuga include 8, 14, 15, 16, 18, 31, and 32, et al. The phenotypes of different species within this genus vary, particularly in flower color and morphology. Investigating how variations in chromosome numbers and corresponding genome sequences within this genus lead to the formation of different species is a valuable research question at the comparative genomic level. Comparing the genomes of different species from the Ajuga genus allows tracing their evolutionary paths. It clarifies the evolutionary history of traits related to medicinal properties, including divergence times and evolutionary pressures. Furthermore, comparative genomics helps identify genes responsible for synthesizing specific bioactive compounds and understanding the biosynthetic pathways of key medicinal compounds. This knowledge can enhance the production of desired compounds in *A. decumbens* plants or model organisms through metabolic engineering. Insights from genomic research can also be used for genetic modification of *A. decumbens* to enhance its stress tolerance and medicinal value. Additionally, genomic data lays the groundwork for synthetic biology, enabling the synthesis of valuable compounds in microbial or other plant systems, offering environmentally sustainable production methods. This research has generated high-quality genomic and transcriptomic data for *A. decumbens*. In-depth studies of the *A. decumbens* genome have deepened our understanding of plant genetics and biochemistry and created tremendous potential for advances in pharmacology, plant biology, biotechnology, and practical applications in medicine and agriculture.

Recent advancements in high-throughput sequencing technologies and decreased costs have shifted the focus from metabolite-based studies to in-depth whole-genome research in various medicinal plants (Chen et al., 2021). Many medicinal plant genomes are highly repetitive or heterozygous, complicating high-quality genome assembly. We employed a hybrid sequencing strategy to achieve the first genome assembly of *A. decumbens*. This genome is substantial (1143 Mb) with 16 pseudochromosome sequences. We annotated 32,452 genes in *A. decumbens*, of which 78.11% were found to be homologous to Arabidopsis sequences, leaving 22.89% as potentially unique to the species or lineage, meriting further investigation. In summary, the assembled *A. decumbens* genome from this study is a valuable resource for genetic research in the Ajuga genus, aiding in identifying significant pharmacologically relevant metabolites.

## Materials and methods

### Plant materials and sequencing

Samples of wild *A. decumbens* for this study were obtained from Nanyang, Henan (33.293° N, 112.024° E) and cultivated under laboratory conditions of 25°C, 3000 lx, and a 16-hour light: 8-hour dark photoperiod. Young leaves were used for genomic DNA extraction, while stems, roots, and leaves were utilized for transcriptome sequencing. These samples were collected from the same individual plant. Immediately after collection, samples were plunged into liquid nitrogen, transferred to the laboratory, and kept at -80°C. High-molecular-weight genomic DNA was prepared using the cetyltrimethylammonium bromide (CTAB) method and purified with the Qiagen genomic kit (Qiagen, 13343). This DNA was used to construct PacBio HiFi sequencing libraries. A 20 kb insert library was created using the SMRTbell template preparation kit (Pacific Biosciences) and sequenced on the PacBio Revio platform in CCS mode. In the Hi-C sequencing procedure, fresh leaf samples underwent formaldehyde treatment for DNA-protein crosslinking. Chromatin was cleaved with MboI, and 5’ overhangs were filled with biotinylated residues. After re-ligation, DNA was broken into roughly 350-bp pieces by sonication. The Hi-C library, made according to standard methods, was sequenced on the DNBSEQ platform in PE150 mode. We prepared a paired-end DNBSEQ library for RNA sequencing with 350-bp inserts using the NextEra DNA Flex Library Prep Kit (Illumina). This was subsequently sequenced on the DNBSEQ-T7 platform (MGI Tech).

### Genome assembly

The *A. decumbens* genome assembly at the contig level was performed using Hifiasm version 0.19.5 (Cheng et al., 2021) with default settings. Raw Hi-C sequencing data were processed using fastp (Chen et al., 2018) version 0.20.1. Cleaned Hi-C paired-end reads were aligned to the assembled genome with Bowtie2 (Langmead and Salzberg, 2012) version 2.3.2 using parameters “–end-to-end –very-sensitive, -L 30”. Hi-C reads alignment to the scaffolds utilized the Juicer pipeline, then processed with 3D-DNA (Dudchenko et al., 2017) using “–editor-repeat-coverage 20” as a parameter. The Juicebox tool (Durand et al., 2016) facilitated assembly visualization. Genome completeness and annotation were assessed with BUSCO (Manni et al., 2021) using the “embryophyta_odb10” configuration file in “genome” mode. Assembly continuity was assessed by calculating the Long Terminal Repeat Assembly Index (LAI) with LTR_retriever (Ou and Jiang, 2018) using default settings.

### Genome annotation

Before gene annotation, EDTA (Ou et al., 2019) version 1.8.4 was used to predict repetitive sequences on the chromosome-level assembly. LTR_Finder (Xu and; Wang, 2007) and LTRharvest (Ellinghaus et al., 2008) were employed to identify LTRs. LTR_retriever (Ou & Jiang, 2018) was used to integrate the results and estimate insertion times. Repeated DNA sequences were predicted using TIR-Learner (Su et al., 2019) and HelitronScanner (Xiong et al., 2014). The final repetitive sequences were annotated using RepeatMasker (Nishimura, 2000).

In gene prediction experiments across 11 species’ genomes, BRAKER3 outperformed Funannotate and MAKER2 (Gabriel et al., 2023a; Gabriel et al., 2023b). BRAKER3 has been successfully applied in gene prediction for approximately 25 species. We harnessed the BRAKER3 pipeline (Gabriel et al., 2023b), integrating RNA-Seq and homology-based approaches for predicting protein-coding genes. First, clean RNA-Seq reads (roots, stems, and leaves) were mapped to the genome using HISAT2 (Kim et al., 2019) (version 2.10.2) to obtain transcriptome mapping data. Protein sequences from OrthoDB (Kriventseva et al., 2019) version 10.0 were downloaded and aligned to the genome assembly using ProtHint version 2.6.0. Then, we used GeneMark-EP+ (Brůna et al., 2020) version 4.65 to integrate the two types of data. Functional annotation of protein-coding genes was conducted via BLASTP (Mahram and Herbordt, 2015), targeting an E-value of < 1e−5 and aligning to prominent public databases such as NCBI NR and Swiss-Prot. Following this, protein domains were identified utilizing InterProScan (Jones et al., 2014) version 4.8. Additionally, EggNOG-mapper (Huerta-Cepas et al., 2019) version 5.0 was employed to determine clusters of orthologous groups (COG).

Non-coding RNAs include rRNAs, tRNAs, snRNAs, and miRNAs. tRNA is predicted using tRNAscan-SE (Chan et al., 2021) version 1.3.1. Due to the high homology of rRNA across different species, rRNA homology searches are performed using Blastn with Arabidopsis rRNA sequences. miRNAs and snRNAs in the genome are identified using INFERNAL (Nawrocki and Eddy, 2013) and PFAM database.

### Phylogenetic analysis

Download the protein sequences of *A. trichopoda*, *O. sativa*, *V. vinifera*, *T. cacao*, *A. thaliana*, *S. lycopersicum*, *C. canephora*, *T. grandis*, *L. japonicus*, *S. miltiorrhiza*, and *A. decumbens* for the following analysis. Orthologous, phylogenetic, and gene family analyses were conducted using OrthoVenn3 (Sun et al., 2023). We used a BLASTP (Mahram and Herbordt, 2015) E-value cutoff of 1E^-5^ and OrthoMCL (Li et al., 2003) Markov clustering to determine pairwise sequence similarity. The phylogenetic tree construction employed FastTree2 (Price et al., 2010) utilizing the JTT+CAT model via the maximum likelihood method, supplemented by the SH test for node reliability assessment. We used single-copy genes and fossil evidence to construct the divergence tree. r8s (Sanderson, 2003) was used to estimate divergence times for *A. thaliana* with *T. cacao*, *S. lycopersicum* with *C. canephora*, *A. thaliana* with *V. vinifera*, *A. trichopoda* with *V. vinifera*, and *L. japonicus* with *T. grandis*. Gene family expansion and contraction were determined using CAFE (Mendes et al., 2020) version 5 by comparing differences in cluster sizes between ancestors and each species. A random birth and death model was used to assess changes in gene families for each lineage in the phylogenetic tree. Conditional likelihood was used as a test statistic, and a P-value equal to or less than 0.01 was considered significant.

### Whole-genome duplication analysis

Protein sequences from *A. decumbens* were analyzed against its protein sequences to identify syntenic blocks. Comprehensive BLASTP analysis, with an e-value threshold of 10^–10^, was employed, followed applying the DupGen_finder pipeline (Qiao et al., 2019) to delineate inter-species syntenic blocks under standard settings. Rates of non-synonymous (Ka) and synonymous (Ks) substitutions, along with their ratio (Ka/Ks), for duplicated gene pairs were computed using the YN model in KaKs_Calculator 2.0 (Wang et al., 2010), following amino acid to codon alignment conversion via PAL2NAL (Suyama et al., 2006) v14.

## Data availability statement

The data presented in the study are deposited in the NCBI repository (PRJNA1042970) and Figshare database (https://doi.org/10.6084/m9.figshare.24596520.v1).

## Author contributions

YG: Writing – review & editing, Writing – original draft. JL: Writing – review & editing, Writing – original draft. YX: Writing – review & editing, Writing – original draft. TZ: Writing – review & editing, Writing – original draft. KT: Writing – review & editing, Writing – original draft. XL: Writing – review & editing, Writing – original draft. LY: Writing – review & editing, Writing – original draft.
