# Supplementary material for: Chromosome-level genome assembly of Ajuga decumbens
Source: Front Plant Sci. 2024 Jun 19;15:1413468. doi: 10.3389/fpls.2024.1413468 (PMC11220202; doi:10.3389/fpls.2024.1413468)
Supplement: Supplementary file 2 [file DataSheet_1.zip › Figure S1 - S7.pdf]

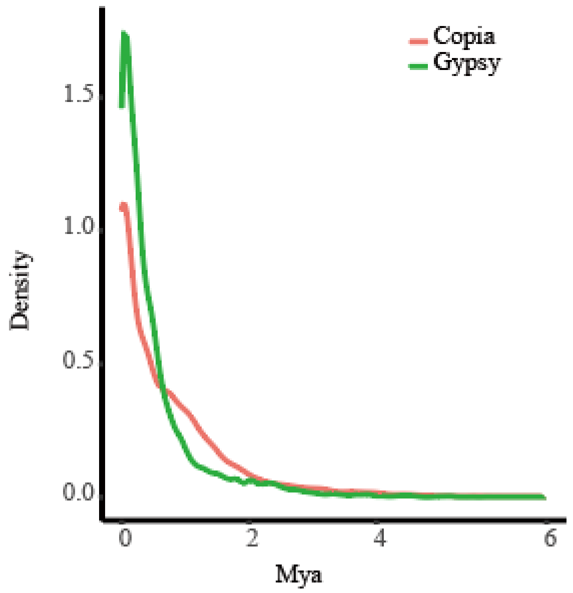

Fig. S1 Density plot showing the burst of long terminal repeats (LTRs) in *A. decumbens*.

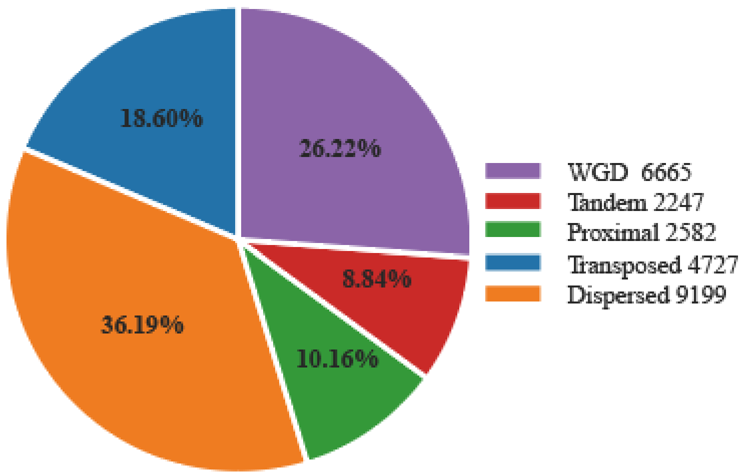

Figure. S2 Pie Chart of Duplicated Genes in *A. decumbens*.

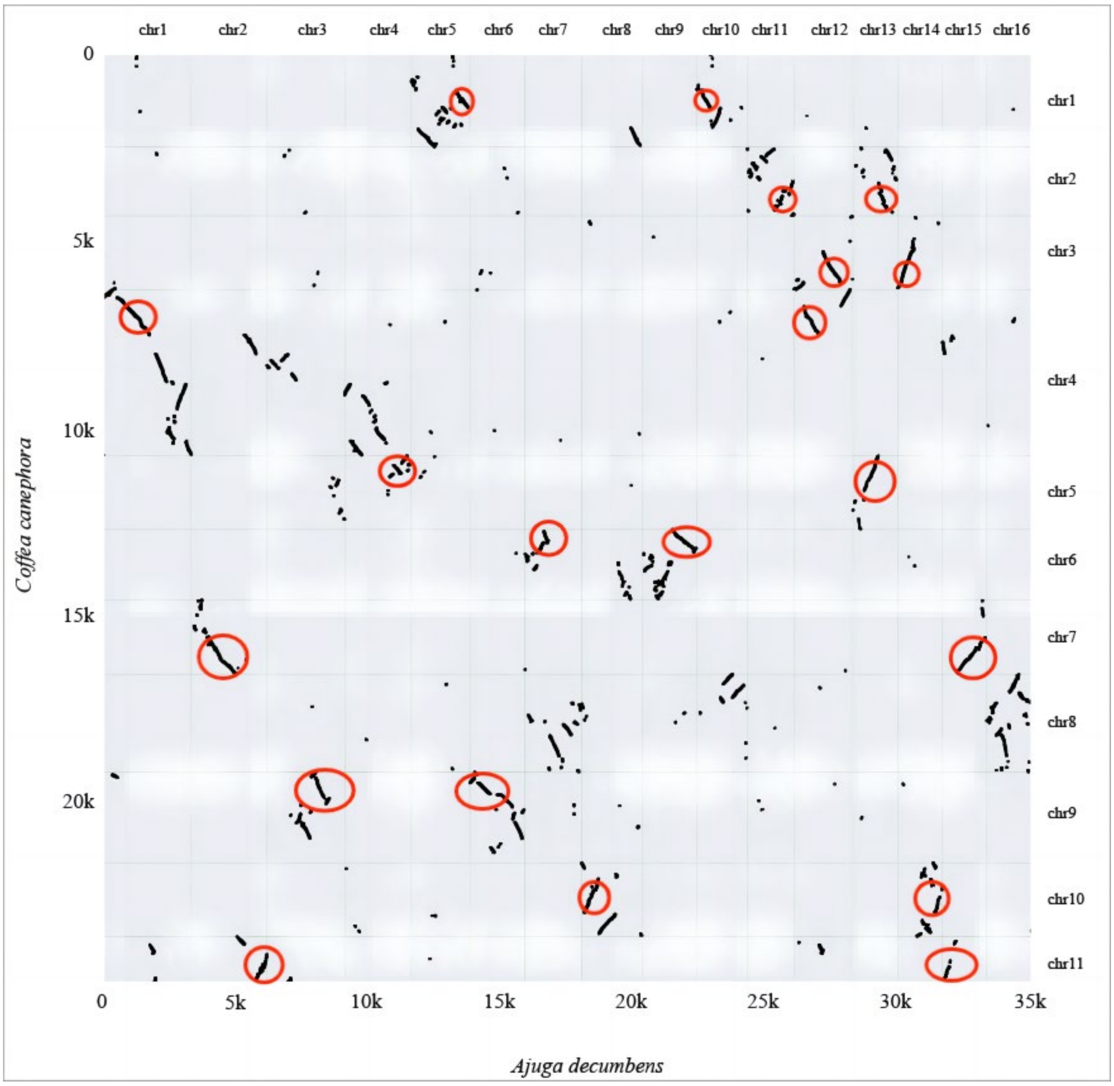

Fig. S3 Homologous dot plot between *A. decumbens* and *C. canephora* chromosomes.

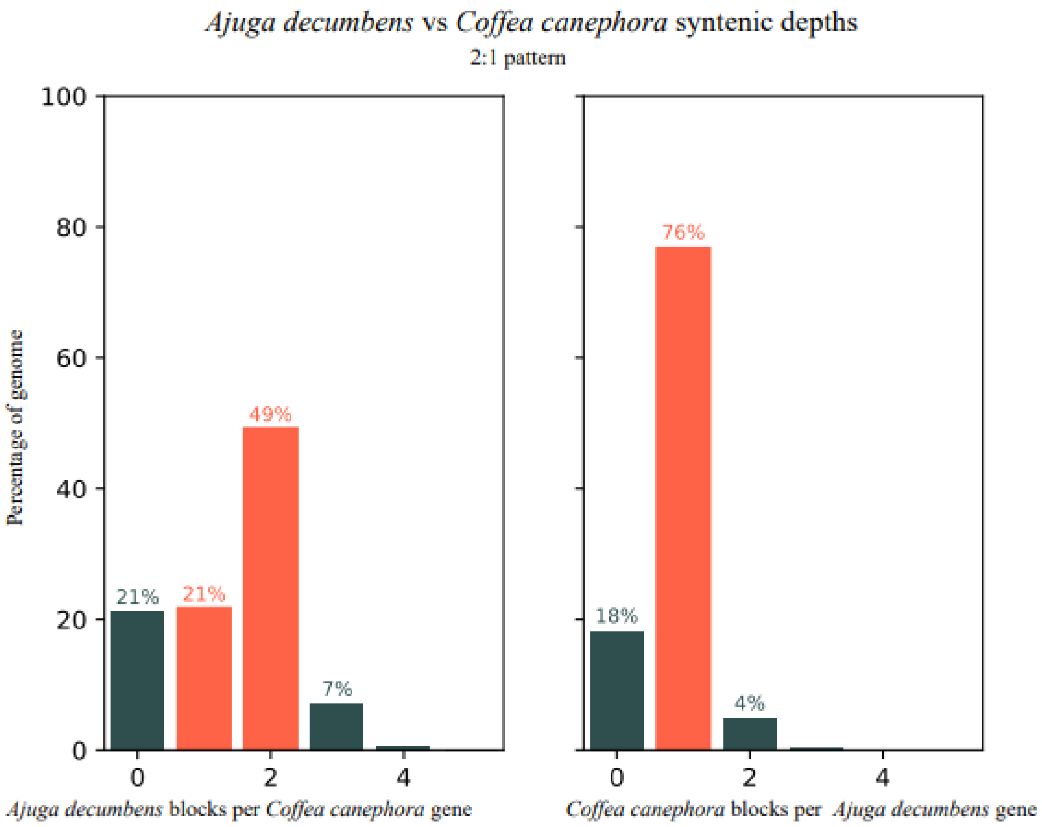

Fig. S4 The synteny pattern between *A. decumbens* and *C. canephora*.

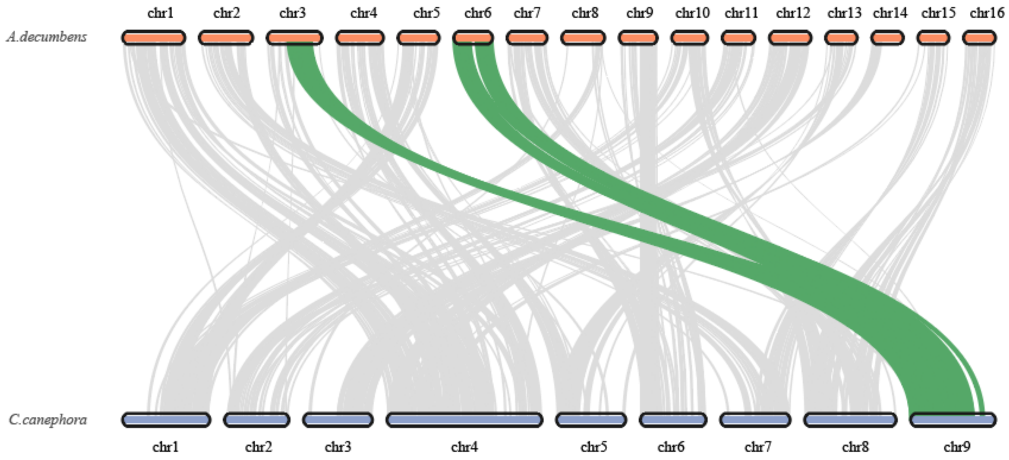

Fig. S5 The synteny blocks between *A. decumbens* and *C. canephora* genomes.

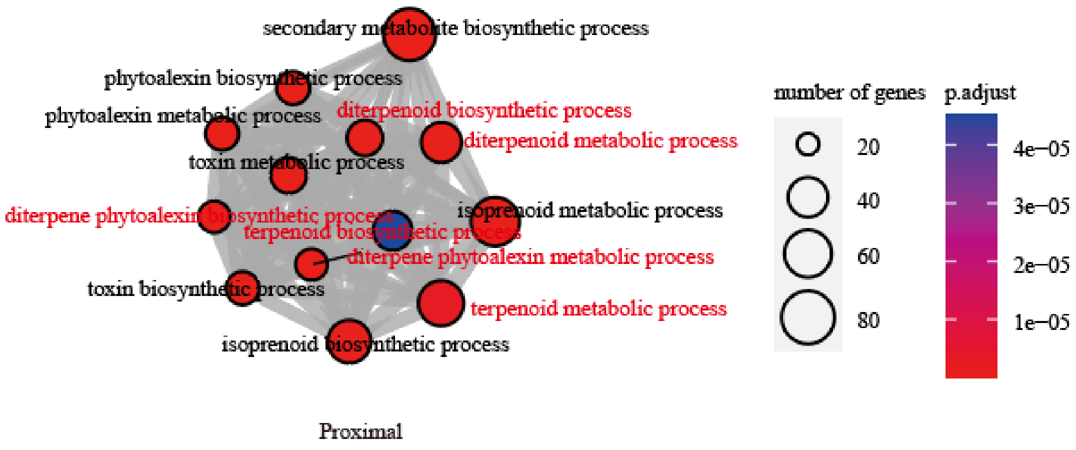

Figure. S6 The cnetplot of GO enrichment associated with terpene synthesis related by proximal genes.

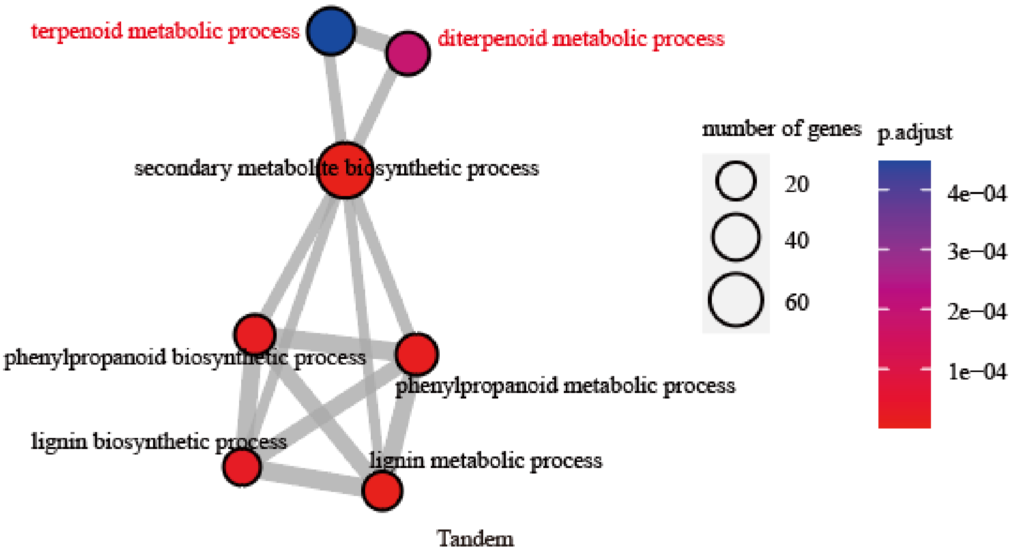

Figure. S7 The cnetplot of GO enrichment associated with terpene synthesis related by tandem genes.
